# Local recurrence and distant metastases 18 years after resection of the greater omentum hemangiopericytoma

**DOI:** 10.1186/1477-7819-5-63

**Published:** 2007-06-06

**Authors:** Maciej Slupski, Ilona Piotrowiak, Zbigniew Wlodarczyk

**Affiliations:** 1 Department of Transplantation and General Surgery, Ludwik Rydygier Collegium Medicum in Bydgoszcz, Nicolaus Copernicus University in Torun, Poland

## Abstract

**Background:**

Hemangiopericytoma occurs with increasing frequency in 5^th ^decade of life and has prediction for retroperitoneum and extremities. A case of a local recurrence and metastases of hemangiopericytoma is described.

**Case presentation:**

Recurrence of hemangiopericytoma in the greater omentum and the jejunal mesentery as well as metastases in the retroperitoneal space were diagnosed in a 61-year-old patient who had a hemangiopericytoma of the greater omentum excised 18 years before.

**Conclusion:**

Because of the rarity of this disease and its typical clinical course associated with late recurrence and metastases, the authors decided to present this case emphasizing the necessity of systematic oncological follow-up after the end of treatment.

## Background

Hemangiopericytoma is a tumor originating from the vascular pericytes of Zimmermann. Most frequently it occurs in patients aged 40 and above with its main localization in the retroperitoneum, pelvis and lower extremities [1-3], however, it may occur at any age in almost any part of body [4]. One of the typical features of this tumor is that both metastasis and recurrence could become apparent even after several years in remission [5-7].

We report the case of a local recurrence and metastases in the retroperitoneum infiltrating the liver and diaphragm in a 61-year-old patient operated on due to a hemangiopericytoma of the greater omentum 18 years before.

## Case presentation

A 61-year-old patient was admitted to the Department of Transplantation and General Surgery with a tumor diagnosed in the left lumbar and retroperitoneal space. Eighteen years before he had been operated for a tumor of the greater omentum with histopathological diagnosis haemangiopericytoma omenti maioris, until now with no recurrence nor distant metastases were found.

On admission the patient was in good general condition with left lumbar pain and a mobile tumor in this area. Abdominal ultrasonography (US) and computed tomography (CT) revealed a retroperitoneum space tumor with suspected infiltration of the segment VII of the liver and diaphragm.

**Figure 1:**
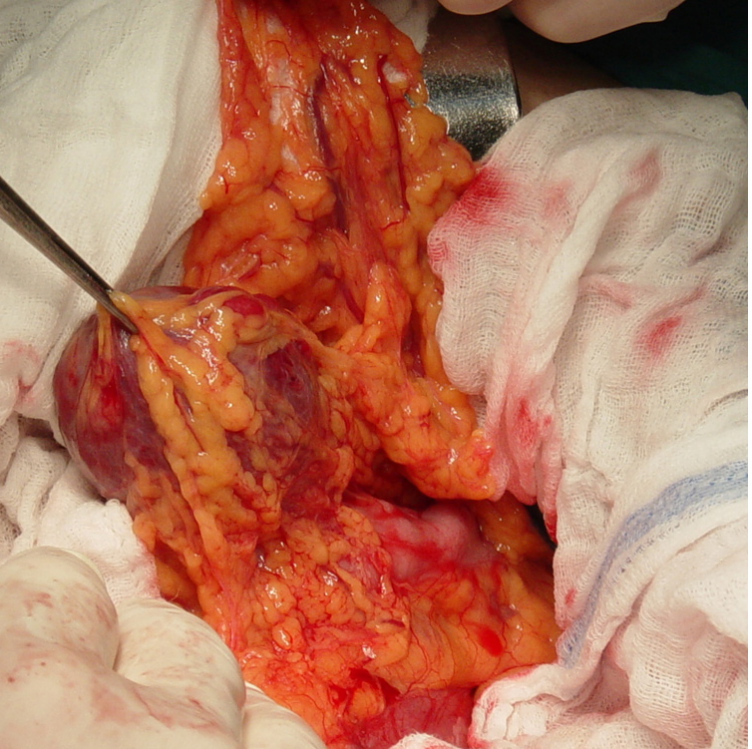
Tumor the greater omentum adjacent to a transverse colon in situ.

**Figure 2:**
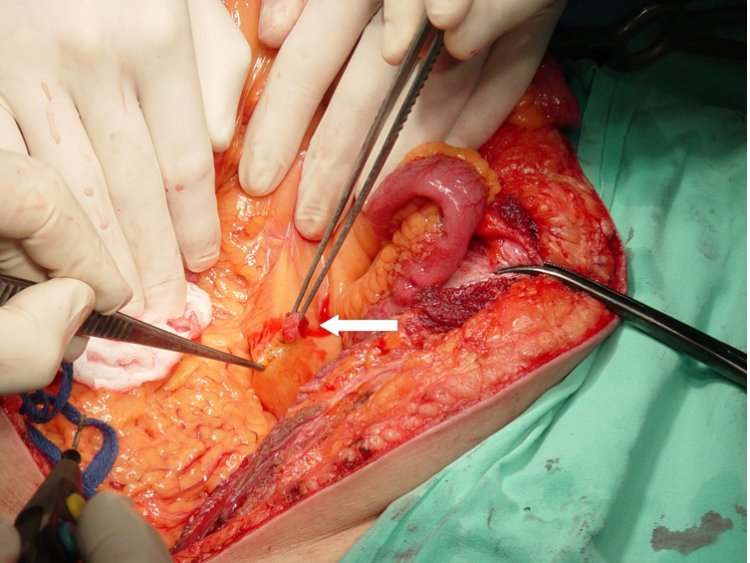
Tumor of the jejunum mesentery (arrow) in situ.

**Figure 3:**
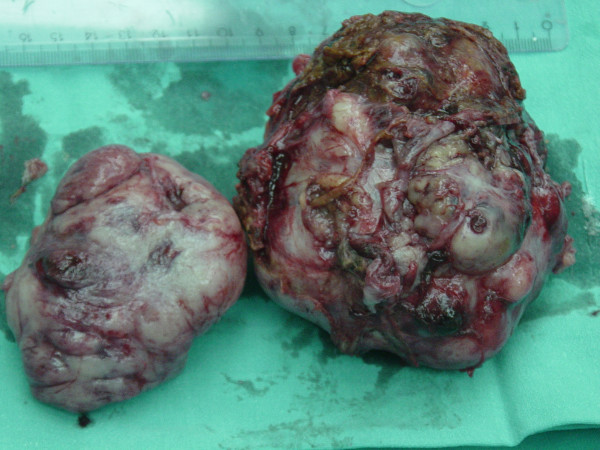
Retroperitoneal space tumor with the infiltrated part of the diaphragm and the segment VII of the liver – postoperative specimen.

**Figure 4:**
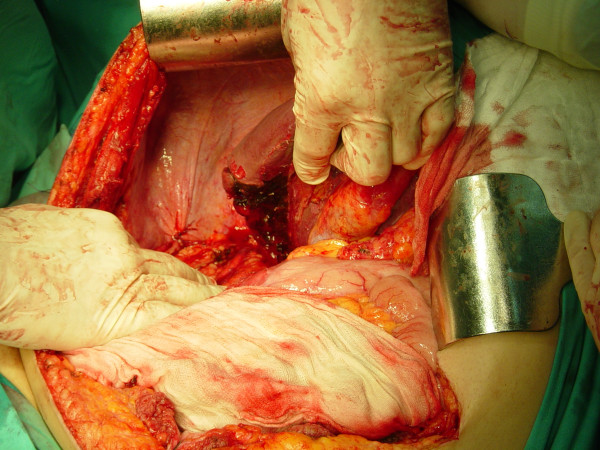
Site of organ removal.

Laboratory investigation showed no abnormalities. The patient was qualified for surgical treatment. A tumor of 5 cm in diameter in the greater omentum tightly adjacent to a transverse colon (figure 1), a tumor of 1 cm in diameter in the jejunum mesentery (figure 2, arrow) and a tumor of 12 cm in diameter located in the retroperitoneal space and infiltrating the diaphragm and the segment VII of the liver were found. No other lesions were found in intraoperative US. The tumors of the greater omentum and of the jejunum mesentery were excised whereas the retroperitoneal space tumor was removed *en bloc *with the infiltrated part of the diaphragm and the segment VII of the liver (figure 3, and 4). The postoperative course proved uneventful. The patient was discharged from the hospital 7 days after the procedure in good general condition with a subsequent surgical follow-up recommended.

Histopathological examination of all the lesions showed haemangiopericytoma malignum. The patient remains under the Oncology Centre and our clinical follow-up and at present, 3 months after the procedure, is in good condition.

## Discussion

Hemangiopericytomas represent less than 1% of all vascular neoplasms [8]. They are highly vascular, therefore could be revealed by angio-CT and angiography (for diagnosis, tumor size, relationship to other viscera, source of blood supply) as well as undergo preoperative transarterial embolization if necessary [3,5]. The tumors' medium size is 6.5 cm and they are encapsulated by a pseudocapsule [1,3]. There is no grading scale for hemangiopericytoma; its malignant potential is implied by histology and clinical behavior [5]. Hypervascularity is a contraindication to a biopsy, therefore a histopathological diagnosis is established after an excision of the lesion. Potentially malignant tumors are characterized by 1 mitotic figure per 10 high-power field and moderate anaplasia. According to McMaster *et al*., 25 of 32 (78%) malignant tumors, 6 of 16 (37.5%) borderline lesions and no benign tumors metastasised [6].

Metastases occur by hematogenous and lymphogenous routes affecting mainly the lung, liver, bones and regional lymph nodes [1,3,9,10]. Metastasis to pleura have also been reported [11]. Metastases occurring many years after excision of a primary lesion is typical of hemangiopericytoma: In our case it was 18 years. Multiple hepatic and bone metastases 12 years after an excision of a nose tumor have been reported [12]. McMaster *et al*., reveal that metastases became apparent in 11% of patients with malignant tumors and 7% with borderline tumors after 5 years' remission [6]. Local recurrence is also common and precedes metastases in more than 2/3 of cases [1,13]. In two cases presented by Panda *et al*., it occurred after 4 months in one patient and 22 years in another [7]. Therefore long-term follow-up is essential [6]. Hypercellularity, mitotic activity, anaplasia, necrosis and hemorrhage are reported to be associated with higher malignant potential [13].

Surgical resection with subsequent radiotherapy remains the treatment of choice for hemangiopericytoma. The role of preoperative angiography and tumor embolization is also emphasized in literature [3,5,6]. Survival rates vary: In a review of 106 cases of hemangiopericytoma, Enzinger *et al*., reported 70% 10-year survival, whereas it was 50% 5-year survival according to other authors [1,10].

Late recurrence is common in other malignancies as well. According to Shen *et al*., in primary cutaneous melanoma the mean disease-free interval after surgery was 182 months [14] whereas Briele *et al*., report seven patients in whom local or regional recurrence occured 11 to 23 years after first treatment of melanoma [15]. Another neoplasm after a treatment of which long-term follow-up is essential is medulloblastoma as recurrences after the Collins' risk period (i.e. age of a patient plus 9 months) have been noticed [16,17]. The average follow-up in case of rectal cancer surgical treatment is 2 years whereas research shows that local recurrence can become evident even after 5.8 years and systemic recurrence after 7.9 years [18]. Continued long-term follow-up in patients treated for above mentioned malignancies proves beneficial [15,17,19].

## Conclusion

Every patient treated due to a malignant or borderline hemangiopericytoma should be under long-term oncological follow-up as the risk of recurrence and distant metastases even after many years is very high.

## Competing interests

The authors declare that they have no competing interests.

## Authors' contributions

**MS **conceived the idea for the study, participated in its design and coordination, analyzed the data, wrote the first draft of the manuscript.

**IP **conducted the literature review, was involved in data collection, analyzed the data, helped to draft the manuscript.

**ZW **participated in the study design and coordination, helped to draft the manuscript.

All authors read and approved the final version of the manuscript.
